# The basic helix-loop-helix transcription factor MdbHLH3 modulates leaf senescence in apple via the regulation of *dehydratase-enolase-phosphatase complex 1*

**DOI:** 10.1038/s41438-020-0273-9

**Published:** 2020-04-01

**Authors:** Da-Gang Hu, Cui-Hui Sun, Quan-Yan Zhang, Kai-Di Gu, Yu-Jin Hao

**Affiliations:** National Key Laboratory of Crop Biology; MOA Key Laboratory of Horticultural Crop Biology and Germplasm Innovation; College of Horticulture Science and Engineering, Shandong Agricultural University, Tai-An, Shandong 271018 China

**Keywords:** Transcriptional regulatory elements, Plant signalling

## Abstract

Basic helix−loop−helix (bHLH) domain-containing transcription factors are known for their roles in regulating various plant growth and developmental processes. Previously, we showed that MdbHLH3 from apple (*Malus domestica*) has multiple functions, modulating both anthocyanin biosynthesis and cell acidification. Here, we show that MdbHLH3 also regulates ethylene biosynthesis and leaf senescence by promoting the expression of *dehydratase-enolase-phosphatase complex 1* (*MdDEP1*). Therefore, we propose a model whereby MdbHLH3 acts as a crucial factor that modulates anthocyanin biosynthesis and cell acidification in addition to fruit ripening and leaf senescence by regulating distinct target genes.

## Introduction

The basic helix−loop−helix (bHLH) superfamily contains transcription factors (TFs) that possess highly conserved alkaline helix-loop-helix domains^1,2^. Regarding the two conserved motifs of a bHLH TF, the basic region is responsible for DNA recognition and binding, whereas the hydrophobic residue-rich HLH region functions in dimerization^3^.

bHLH TFs have been widely studied in many animal and plant species. Previous studies have demonstrated that bHLH TFs play roles as key regulators of various plant growth and development processes. In apple (*Malus domestica*), MdbHLH3 has been identified as a multifunctional regulator. It was initially characterized by Xie et al.^4^ as an anthocyanin biosynthesis regulator. MdbHLH3 interacts with the MYB TF MdMYB1 with which it forms a complex to directly activate the downstream anthocyanin biosynthesis genes *MdDFR* and *MdUFGT* at low temperatures, thereby promoting anthocyanin accumulation and fruit coloration in apple. In addition, a previous study found that the glucose sensor MdHXK1 phosphorylates MdbHLH3, leading to enhanced transcription of downstream anthocyanin biosynthesis genes and increased anthocyanin accumulation^5^. MdbHLH3 was also characterized as a crucial component of the MYB-bHLH-WDR (MBW) complex, which controls vacuolar transport of anthocyanins^6^. Recently, we reported that the glucose-inhibited ubiquitin E3 ligase MdPUB29 ubiquitinates MdbHLH3 to influence ethylene biosynthesis and fruit ripening^7^. Overall, MdbHLH3 is considered a multifunctional regulator in apple that, in addition to regulating anthocyanin biosynthesis, is involved in processes such as environmental responses, cell acidification, ethylene biosynthesis, sugar homeostasis, and phytohormone signaling pathways.

Here, we report that apple dehydratase-enolase-phosphatase complex 1 (MdDEP1), initially characterized as active in the recycling of the ethylene precursor S-adenosylmethionine (SAM), is essential for regulating leaf senescence. Furthermore, MdbHLH3 was shown to mediate leaf senescence via direct activation of *MdDEP1* expression. The exploitation of the multiple MdbHLH3-mediated functions in breeding programs is discussed.

## Materials and methods

### Plant materials and growth conditions

Tissue culture plantlets of the apple (*Malus domestica*) ‘Gala’ cultivar were maintained in vitro on MS medium supplemented with 0.2 mg L^−1^ IAA and 0.8 mg L^−1^ 6-BA, with subculturing performed once a month. The plantlets were grown at room temperature under long-day conditions (16-h light/8-h dark). Calli induced from young embryos of ‘Orin’ apple (*Malus domestica* Borkh.) were proliferated at room temperature in darkness on MS medium supplemented with 1.5 mg L^−1^ 6-BA and 0.5 mg L^−1^ IAA. The calli were subcultured at a minimum average of 5 day intervals in preparation for their use in genetic transformations and GUS assays.

### Gene expression analysis

RNA extraction, cDNA synthesis, and quantitative real-time PCR (qRT-PCR) assays were carried out as previously described^6^. All study-associated primer sequences are listed in Supplementary Table 1.

### Construction of plasmids and genetic transformation

The full-length *MdDEP1* ORF was amplified by PCR and introduced into the pCXSN::Myc vector to overexpress *MdDEP1* under the *35S* promoter. The resulting construct was introduced into the maintained tissue culture of ‘Gala’ plantlets described above via *Agrobacterium* (LBA4404)-mediated transformation as described previously^6^.

For ‘Orin’ calli transformation, the P_MdDEP1_::udiA construct was used to transform the calli following an *Agrobacterium*-mediated method as described by Hu et al.^7^.

### Chromatin immunoprecipitation (ChIP) qPCR and electrophoretic mobility shift assay (EMSA) analyses

The calli expressing *35S::Myc* and *35S::MdbHLH3-Myc* were subjected to ChIP-qPCR analysis. We used an anti-Myc antibody (Beyotime)^6^. The immunoprecipitated DNA samples were used as templates for qPCR assays.

EMSA was conducted as described by Hu et al.^6^. MdbHLH3-His recombinant protein was expressed in *Escherichia coli* strain BL21 and purified using glutathione sepharose beads (Thermo Scientific, San Jose, CA, USA). The EMSA probe biotin labeling kit (Beyotime) was used to label an oligonucleotide sequence corresponding to the *MdDEP1* promoter, which was subsequently used for the binding assays with a LightShift chemiluminescent EMSA kit (Thermo).

### GUS expression analysis

GUS reporter constructs were created containing the promoter sequence of *MdDEP1*, with cloning performed as previously described^7^.

### Chlorophyll content determination

Determination of the chlorophyll content was performed as previously described^7^. The apple leaves used for chlorophyll content determination were obtained from five different developmental stages (T0, T1, T2, T4, and T6). These five stages were defined according to the characteristics of leaf age in reference to the second leaf from the bottom of the apple shoot (T0: 20 days; T1: 30 days; T2: 40 days; T4: 50 days; and T6: 60 days).

### Statistical analysis

At least three biological replicates were included for all samples, and the data are presented as the means ± standard deviation unless otherwise indicated. Significant differences were determined with Student’s *t*-test (*p* ≤ 0.01, significantly different; *p* ≤ 0.001, very significantly different).

## Results

### MdbHLH3 influences leaf senescence in apple

**Fig. 1:**
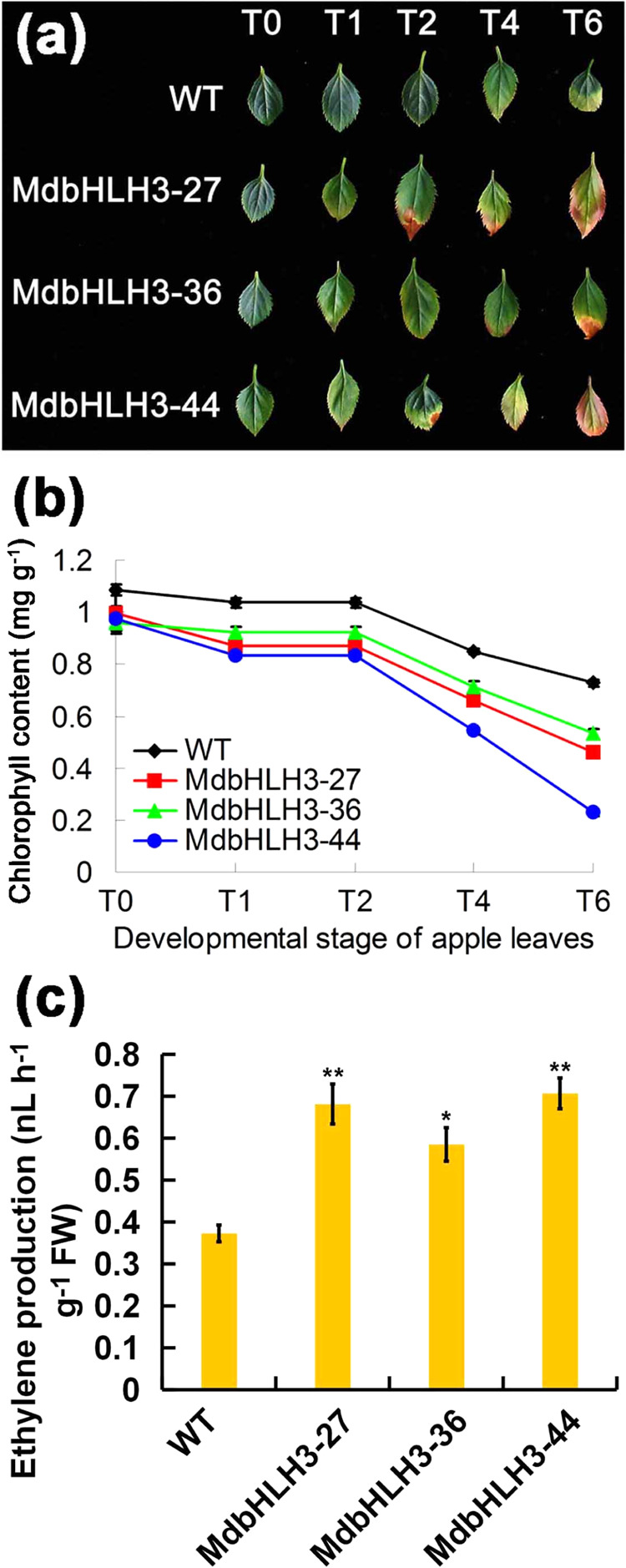
MdbHLH3 controls leaf senescence in apple. **a** Leaf senescence phenotype in the WT and three *35S::MdbHLH3* transgenic apple plantlets. Note: T0 to T6 represent the leaves in different developmental stages. **b** Chlorophyll content of the leaves shown in **a**. **c** Ethylene production in the WT and three *35S::MdbHLH3* transgenic apple plantlets. The data represent the means ± SE of three independent experiments. Significance was determined using Student’s *t*-test; ^*^P < 0.01 and ^**^P < 0.001

Three apple lines harboring the *35S::MdbHLH3-GFP* construct^4^ were further investigated. Phenotypic analysis of these lines showed that, compared to that of the wild-type (WT) control, *MdbHLH3* overexpression was clearly associated with more severe leaf senescence symptoms starting at the T2 developmental stage (Fig. 1a), which became more severe in later stages. Moreover, faster chlorophyll degradation was observed in the *MdbHLH3*-overexpressing lines than in the WT control (Fig. 1b), and the transgenic lines produced a higher level of ethylene than did the control (Fig. 1c). Combined, these results point to a key role of *MdbHLH3* in leaf senescence.

### MdbHLH3 binds to the *MdDEP1* promoter

To investigate the potential downstream target genes regulated by MdbHLH3, ChIP-seq combined with targeted sequence capture was performed. Among the identified candidates, MDP0000180420 encodes dehydratase/enolase/phosphatase MdDEP1, which functions in methionine salvaging (Yang cycle). The plant-specific DEP1 enzyme is well known for its trifunctional dehydratase, enolase, and phosphatase activities and converts 5-methylthioribulose-1-P (MTRu-1-P) directly to 1,2-dihydroxy-3-keto-5-methylthiopentene (DHKMP), the reciprocal third compound in the methionine salvage pathway^8^. *MdDEP1* was identified through cDNA-AFLP, which is associated with low fruit acidity in apple^9^.

**Fig. 2:**
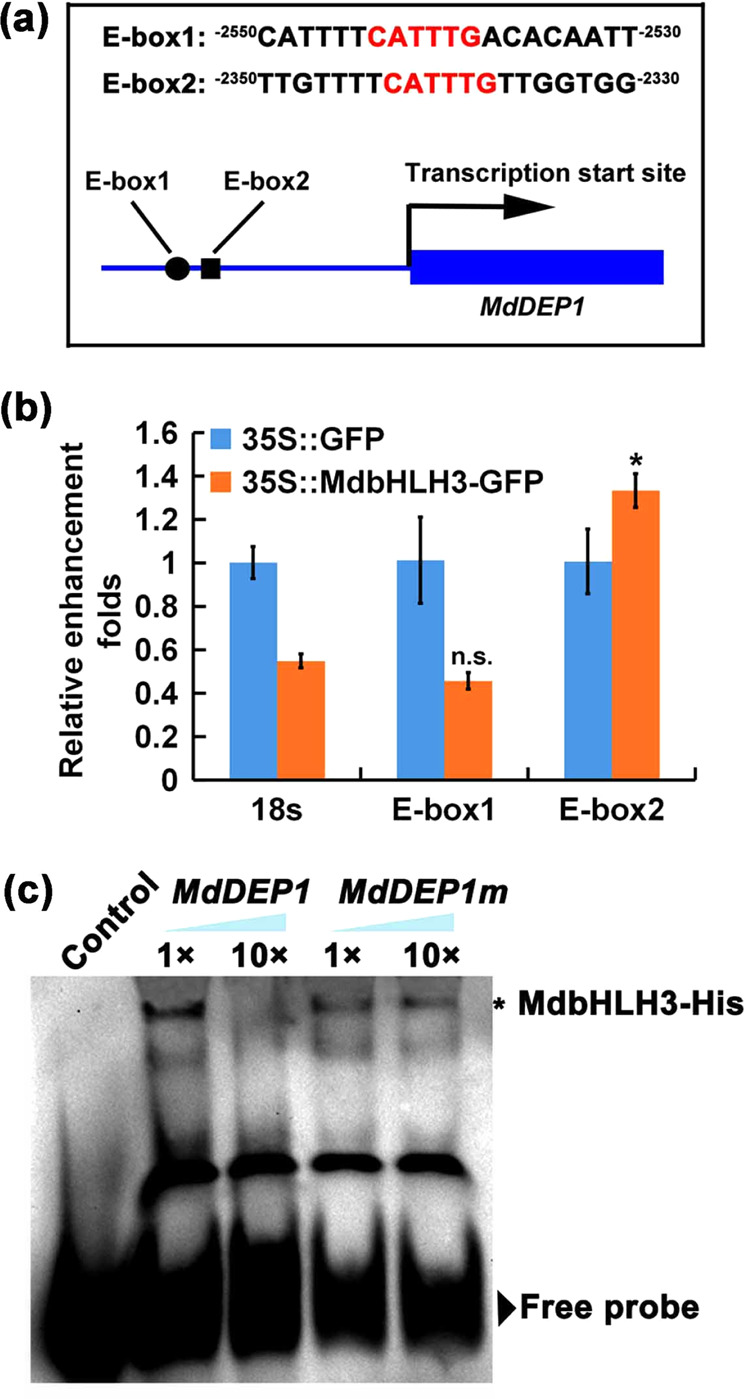
MdbHLH3 binds to the *MdDEP1* promoter. **a** The putative MdbHLH3 TF-binding element in the *MdDEP1* promoter. The black box and cycle represent the two MdbHLH3-binding motifs. **b** The relative enrichment of the *MdDEP1* gene promoter fragments. The MdbHLH3-DNA complex was coimmunoprecipitated from *35S::MdbHLH3-GFP*-transfected apple leaf protoplasts using an anti-GFP antibody. 35S::GFP was used as a negative control. **c** Results from the EMSA assays of the interaction between MdbHLH3 and labeled E-box2 DNA probes in the *MdDEP1* promoter

To confirm the role of MdbHLH3 in *MdDEP1* gene expression regulation, we analyzed *cis*-elements within the *MdDEP1* promoter region, which revealed two typical bHLH-binding E-boxes (5′-CATTTG-3′) (Fig. 2a). Chromatin immunoprecipitation PCR (ChIP-PCR) assays were carried out on apple leaf protoplasts individually transfected with the *35S::MdbHLH3-GFP* construct or the *35S::GFP* construct. The bHLH element-containing E-box2 region from the *MdDEP1* promoter, but not the E-box1 region, was enriched in the ChIP protoplasts transfected with *35S::MdbHLH3-GFP* compared with the protoplasts transfected with the *35S::GFP* control (Fig. 2b).

To confirm the direct binding of MdbHLH3 and the *MdDEP1* promoter, the MdbHLH3-His fusion protein was subjected to an in vitro electrophoretic mobility shift assay (EMSA). We detected a specific DNA-MdbHLH3 protein complex using a probe representing the CATTTG-containing E-box2 sequence (Fig. 2c). Accumulation of this complex was reduced in proportion to the amount of unlabeled competitor probe with the same sequence included in the assay, whereas competition was absent in the assay with a probe comprising a different sequence (Fig. 2c). These results indicate that MdbHLH3 specifically binds to the bHLH recognition sequence in the *MdDEP1* promoter.

### MdbHLH3 transcriptionally activates and positively regulates *MdDEP1* gene expression

**Fig. 3:**
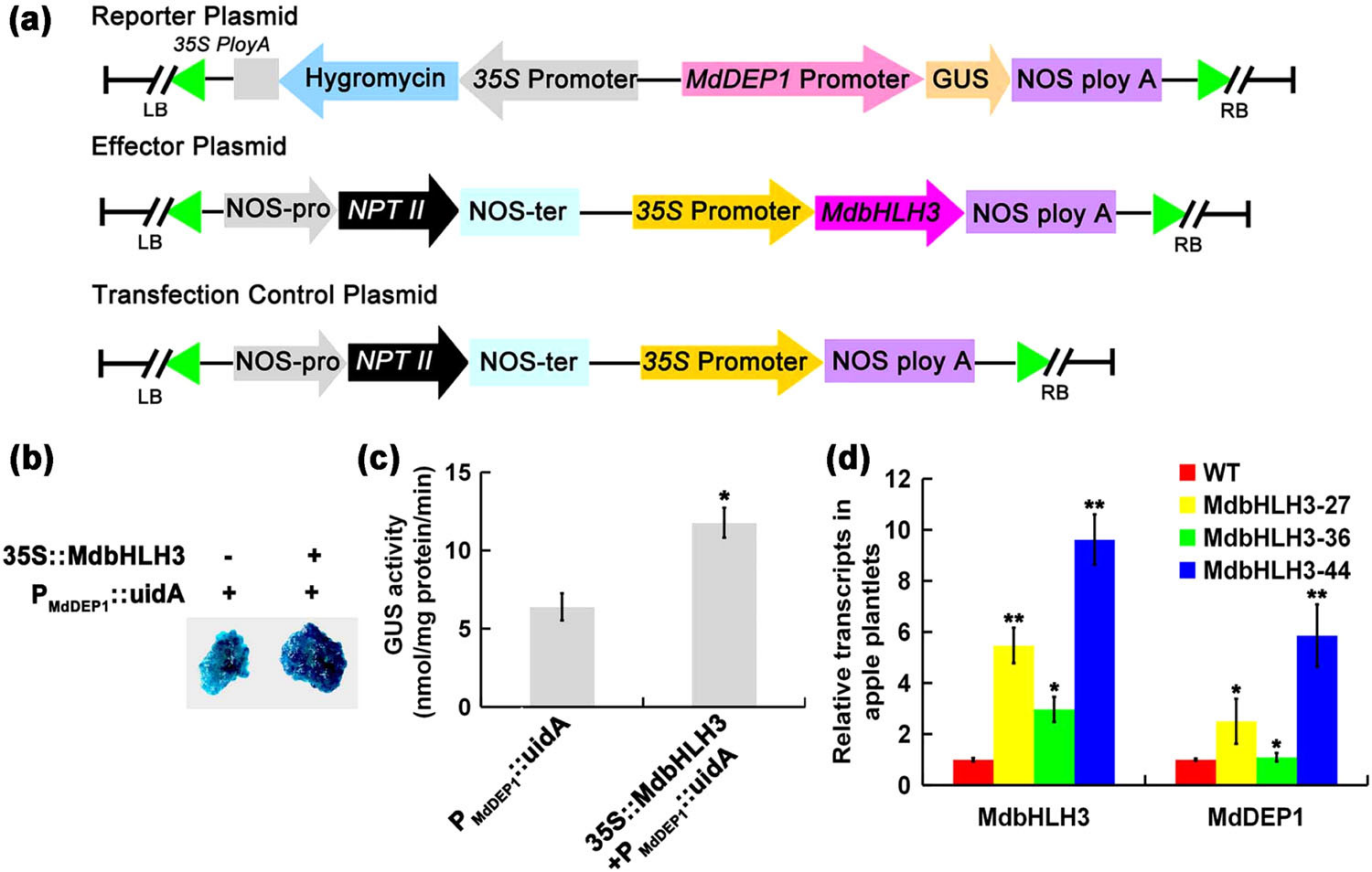
MdbHLH3 transcriptionally activates and positively regulates *MdDEP1* gene expression. **a** The effector and reporter constructs in the binary vectors were introduced into apple calli for the GUS activity assays. **b** *P*_*MdDEP1*_*::uidA* transgenic apple calli with or without the 35S::MdbHLH3 effector were grown at 25 °C in the dark and stained to visualize the GUS activity. **c** GUS activity in the transgenic apple calli as labeled. **d** Relative transcripts of *MdbHLH3* and *MdDEP1* in transgenic apple lines overexpressing *MdbHLH3* (MdbHLH3-27, MdbHLH3-36, MdbHLH3-44) and the WT control. The data represent the means ± SE of three independent experiments. Statistical significance was determined using Student’s *t*-test; ^*^P < 0.01 and ^**^P < 0.001

GUS assays were performed to verify the transcriptional activation of *MdDEP1* by MdbHLH3. Calli were transformed with the construct *P*_*MdDEP1*_*::udiA* alone or in combination with the *35S::MdbHLH3* construct. Transgenic calli expressing *P*_*MdDEP1*_*::uidA* and *35S::MdbHLH3* exhibited much higher GUS enzyme activity than those expressing *P*_*MdDEP1*_*::uidA* alone (Fig. 3a–c), thus indicating that MdbHLH3 enhanced GUS expression by interacting with the *MdDEP1* promoter. The results from the qRT-PCR analysis showed that the transgenic *MdbHLH3*-overexpressing plantlets possessed a higher level of *MdDEP1* transcript than was expressed in the WT plantlets (Fig. 3d), confirming that *MdDEP1* expression was positively regulated by MdbHLH3.

### MdDEP1 regulates leaf senescence in apple

**Fig. 4:**
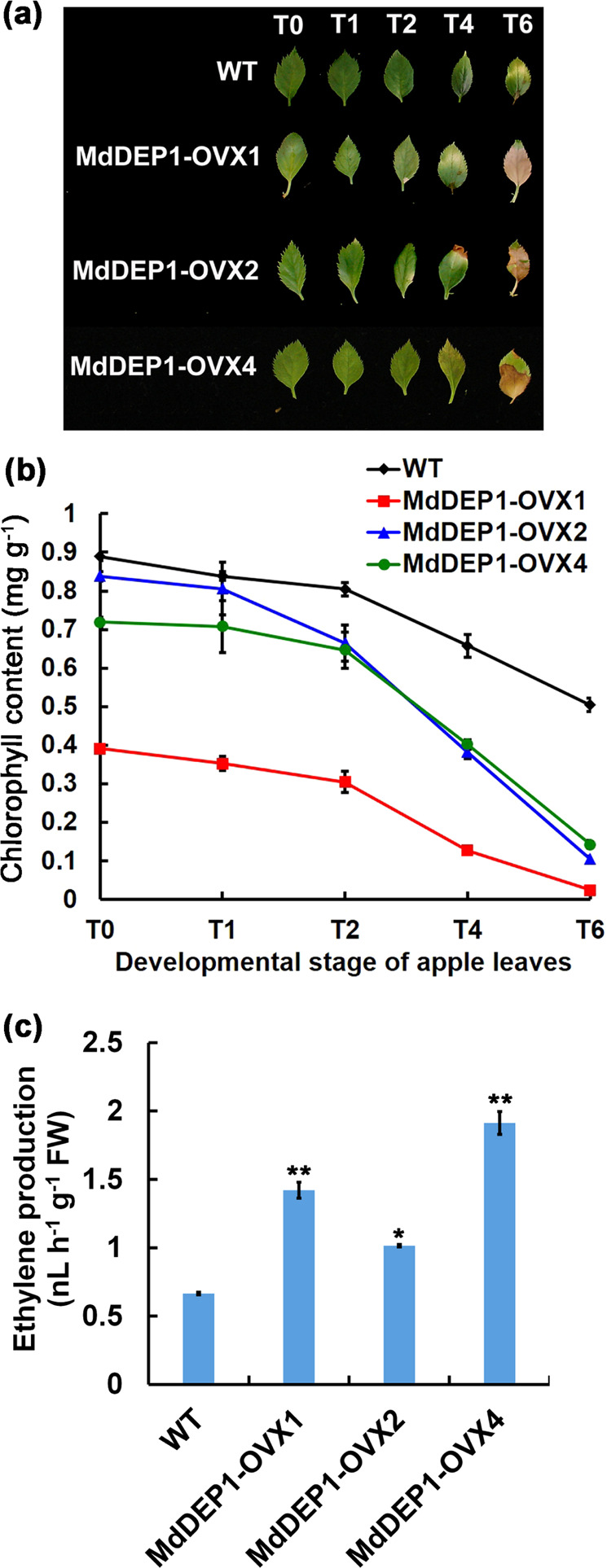
MdDEP1 regulates leaf senescence in apple. **a** Leaf senescence phenotype in the WT and three *35S::MdbHLH3* transgenic apple plantlets. Note: T0 to T6 represent the leaves in different developmental stages. **b** Chlorophyll content of the leaves shown in **a**. **c** Ethylene production in the WT and three *35S::MdDEP-Myc* transgenic apple plantlets. The data represent the means ± SE of three independent experiments. Statistical significance was determined using Student’s *t*-test; ^*^P < 0.01 and ^**^P < 0.001

Since MdbHLH3 modulates leaf senescence and positively regulates *MdDEP1* gene expression, it is reasonable to assume that MdDEP1 influences leaf senescence. To further validate the role of *MdDEP1* in leaf senescence, a Myc-tagged MdDEP1 fusion protein with expression driven by the *35S* promoter (*35S::MdDEP1-Myc*) was expressed in apple plants. Several independent transgenic lines were identified by qRT-PCR analysis (Supplementary Fig. 1), of which three (MdDEP1-OVX1, MdDEP1-OVX2, and MdDEP1-OVX4) exhibited higher *MdDEP1* expression levels. In addition to enhanced ethylene production, these *MdDEP1*-overexpressing lines showed more severe leaf senescence symptoms compared to those shown in the WT control, commencing at the T2 developmental stage (Fig. 4a–c). At later developmental stages, leaf yellowing and the degradation of chlorophyll were further enhanced in the *MdDEP1*-overexpressing lines (Fig. 4a, b). These results support the conclusion that *MdDEP1* also plays a key role in leaf senescence in apple.

## Discussion

The Yang cycle indicates that 5’-methylthioadenosine (MTA) is recycled during the production of methionine from ethylene, nicotianamide, or polyamines^8^. This cycle plays important roles in ethylene-mediated leaf senescence^8,10^. Among those enzymes active in the Yang cycle, the plant-specific enzyme DEP1 possesses phosphatase, enolase, and dehydratase activities and converts 5-methylthioribulose-1-P (MTRu-1-P) directly to 1,2-dihydroxy-3-keto-5-methylthiopentene (DHKMP), the reciprocal third compound in this pathway^8^. The present study provides evidence that MdbHLH3 controls leaf senescence by directly activating *MdDEP1* expression in apple.

**Fig. 5:**
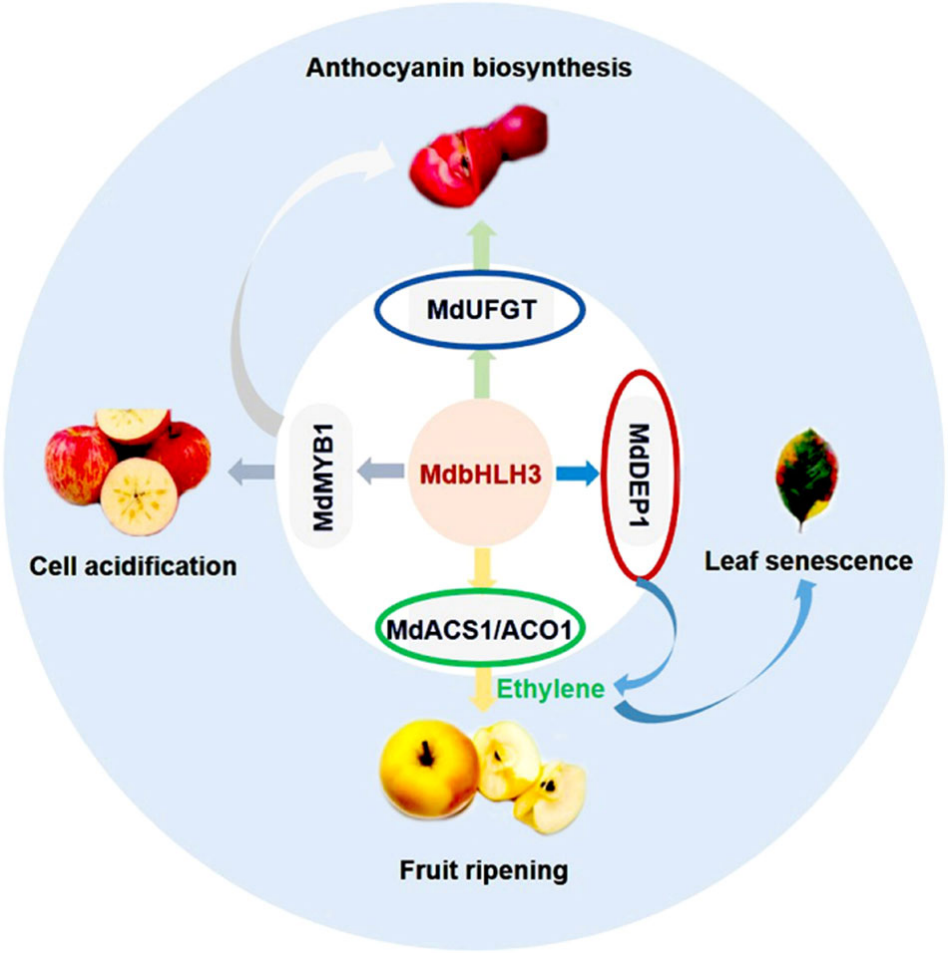
Model demonstrating the multifaceted roles of MdbHLH3 in apple. MdbHLH3 interacts with the MYB TF MdMYB1, forming a complex that directly activates the downstream anthocyanin biosynthetic *MdUFGT* gene, thereby enhancing anthocyanin content and fruit coloration; MdbHLH3 controls the expression levels of genes encoding vacuolar transporters, including *MdVHAs*, *MdVHP1*, and *MdtDT*, resulting in vacuolar acidification and anthocyanin accumulation; MdbHLH3 controls the ethylene level by directly activating the expression of the ethylene biosynthetic genes *MdACO1*, *MdACS1*, and *MdACS5A7*; and MdbHLH3 modulates leaf senescence by directly activating *MdDEP1* expression

DEP1 is a plant-specific trifunctional enzyme that converts MTRu-1-P directly to KMTB in *Arabidopsis*^8^. Consistent with its role in the Yang cycle, apple plantlets overexpressing *MdDEP1* accumulated a significantly higher amount of methionine (Supplementary Fig. 2). This accumulation may explain the higher ethylene production observed in the *MdDEP1* transgenic plantlets compared to the level produced in the WT control (Fig. 4c). On the other hand, our previous study showed that MdbHLH3 controls ethylene levels by directly activating the expression of the ethylene biosynthetic genes *MdACO1*, *MdACS1*, and *MdACS5A*^7^. These results suggest that MdbHLH3 either directly or indirectly controls ethylene production underlying leaf senescence through multiple biological pathways (Fig. 5).

Additionally, MdbHLH3 interacts with the MYB TF MdMYB1, forming a complex that directly activates *MdDFR* and *MdUFGT*, downstream anthocyanin biosynthetic genes, in apple^4^ at low temperatures, thereby enhancing anthocyanin content and fruit coloration. Moreover, the glucose sensor MdHXK1 phosphorylates MdbHLH3, resulting in enhanced transcription of downstream anthocyanin biosynthesis genes and increased anthocyanin accumulation^5^. MdbHLH3 was also shown to be a crucial component of the MYB-bHLH-WDR (MBW) complex that controls expression levels of genes encoding vacuolar transporters, including MdVHAs, MdVHP1, and MdtDT, resulting in vacuolar acidification and anthocyanin accumulation^6^.

Finally, our laboratory recently demonstrated a role for MdbHLH3 in fruit ripening and ethylene biosynthesis upon ubiquitination of the ubiquitin E3 ligase MdPUB29, which can be inhibited by glucose^7^. Combined, these findings support the notion that MdbHLH3 is a multifunctional regulator in apple and is involved in many other processes aside from anthocyanin biosynthesis, such as environmental responses, cell acidification, ethylene biosynthesis, sugar homeostasis, and phytohormone signaling pathways. Therefore, it is reasonable to conclude that MdbHLH3 acts as a multifaceted regulator that functions in anthocyanin biosynthesis, cell acidification, fruit ripening, and leaf senescence by regulating different target genes (Fig. 5).

Leaf senescence is a complicated and crucial physiological process in plants^8,10^. Elucidating this mechanism is a key step to understanding a series of biological phenotypes^11^. Additionally, leaf senescence is a major target of breeding programs for many crop plants. Our findings add to the current understanding of how the MdbHLH3-MdDEP1 regulatory module affects leaf senescence. Taken together, these results describe a novel molecular mechanism of these important processes in plants. The results of this study may also contribute to the development of biotechnological approaches and tools for future phytoremediation applications.

## Supplementary information

Supplemental Information
