# Supplementary material for: The basic helix-loop-helix transcription factor MdbHLH3 modulates leaf senescence in apple via the regulation of dehydratase-enolase-phosphatase complex 1
Source: Hortic Res. 2020 Apr 1;7:50. doi: 10.1038/s41438-020-0273-9 (PMC7109056; doi:10.1038/s41438-020-0273-9)
Supplement: Supplementary file 1 — Supplemental Information [file 41438_2020_273_MOESM1_ESM.docx]

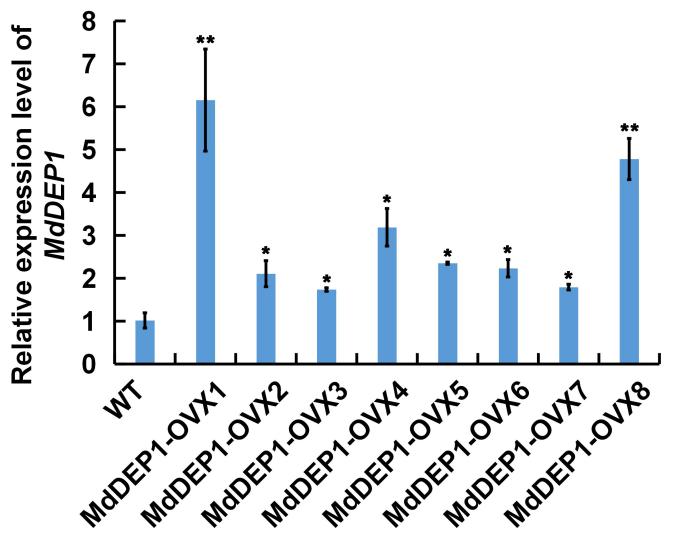


**Supplementary Fig. 1** **The expression of *MdDEP1* in the WT and *35S::MdDEP1-Myc* transgenic apple plants by qRT-PCR analysis.** The reference gene *18s* was used as control.


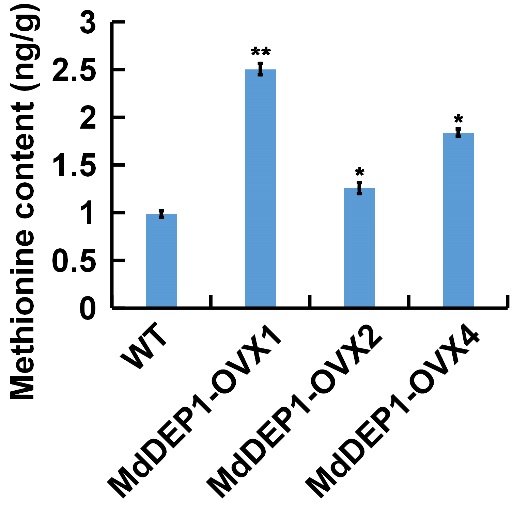


**Supplementary Fig. 2** **The methionine content in WT and three *MdDEP1* transgenic apple leaves**. The data represent the means± SE of three independent experiments. Statistical significance was determined using a Student's *t*-test. *: P < 0.01; **: P < 0.001.

**Supplementary Table 1** The primers used for RT-PCR and qRT-PCR in this study.

| **Name** | **Primer sequences** | **Note** |
| --- | --- | --- |
| MdDEP1-F(+A) | AATGGCAGCGGAAGCTGAGGTTC | Full length primers of *35S::MdDEP1-GFP/35S::MdDEP1-Myc* |
| MdDEP1-R(+A) | TATCATGCTAGCCTCTTCTTTTAG |  |
| pMdDEP1-F | GTAACATCTCACATCAACCAACG | The primers of *MdDEP1* promoter |
| pMdDEP1-R | TTGCCACAGTCGTATGATTCATC |  |
| MdDEP1-F(chip)-cis1 | AGCGGTTTCATCTCATTTT | ChIP-PCR primers |
| MdDEP1-R(chip)-cis1 | ATATACGCCGTCCAAATAGC |  |
| MdDEP1-F(chip)-cis2 | GCATGGTACTCTTTCGGTC |  |
| MdDEP1-R(chip)-cis2 | TCATCTTTTCTCCACCAAC |  |
| MdDEP1-F(EMSA-box2) | GTTTCGTAAAATTTGTTTTCATTTGTTGGTGGAGAAAAG | EMSA primers |
| MdDEP1-R(EMSA-box2) | CTTTTCTCCACCAACAAATGAAAACAAATTTTACGAAAC |  |
| mMdDEP1-F(EMSA-box2) | GTTTCGTAAAATTTGTTTTTTTGCATTGGTGGAGAAAAG |  |
| mMdDEP1-R(EMSA-box2) | CTTTTCTCCACCAATGCAAAAAAACAAATTTTACGAAAC |  |
| P_MddDEP1_::udiA-F | GTAACATCTCACATCAACCAACG | GUS vector primers |
| P_MdDEP1_::udiA-R | TTGCCACAGTCGTATGATTCATC |  |
| 18S-F | TGACCGAATGAGCAAGGAAATTACT | qPCR primers |
| 18S-R | TACTCAGCTTTGGCAATCCACATC |  |
| MdDEP1-F | TGAAAGCAAGAACGGAATCTCA |  |
| MdDEP1-R | CCTTCACTATGTTCCTCCACCAC |  |
